# Cancer risk among individuals with mental illness – a systematic review and meta-analysis of nordic register-based prospective studies

**DOI:** 10.1038/s41398-026-04351-5

**Published:** 2026-08-01

**Authors:** Xue Xiao, Fatemeh Sadeghi, Gillian Murphy, Zhe Zhang, Ole A. Andreassen, Unnur Valdimarsdóttir, Fang Fang, Mary M. Barker

**Affiliations:** 1 Department of Otolaryngology-Head & Neck Surgery, First Affiliated Hospital of Guangxi Medical University, Nanning, Guangxi China; 2 Key Laboratory of Early Prevention and Treatment for Regional High-Frequency Tumor (Guangxi Medical University), Ministry of Education & Guangxi Key Laboratory of Early Prevention and Treatment for Regional High Frequency Tumor (Guangxi Medical University), Nanning, Guangxi China; 3 Department of Epidemiology and Biostatistics, School of Public Health, Imperial College London, London, UK; 4 Unit of Integrative Epidemiology, Institute of Environmental Medicine, Karolinska Institutet, Stockholm, Sweden; 5 NORMENT Centre, Division of Mental Health and Addiction, University of Oslo, Oslo, Norway; 6 Oslo University Hospital, Oslo, Norway; 7 Center of Public Health Sciences, Faculty of Medicine, University of Iceland, Reykjavík, Iceland; 8 Department of Epidemiology, Harvard T. H. Chan School of Public Health, Boston, MA USA

**Keywords:** Psychiatric disorders, Schizophrenia

## Abstract

Although cancer patients have a high burden of mental illness, it is less clear whether patients with mental illness experience a higher-than-expected cancer risk. This systematic review investigated the associations between a wide range of types of mental illness and subsequent cancer risk. Five electronic databases were searched for articles published from database initiation to October 24, 2024. Studies using nationwide Nordic register data to conduct a prospective analysis of the association between mental illness diagnosis and cancer incidence were included. When data from three or more countries were available for a given type of mental illness, random effects meta-analyses were performed; otherwise, narrative syntheses were conducted. A total of 7,767 non-duplicate articles were screened, of which 20 were included. Meta-analyses showed no association between schizophrenia diagnosis and overall cancer risk; however, a high level of heterogeneity was detected between studies. Individuals with schizophrenia appeared to have a higher risk of breast cancer (pooled standardized incidence ratio [SIR]: 1.28, 95% confidence interval [CI]: 1.08-1.52), and lower risks of rectal cancer (pooled SIR: 0.75, 95% CI: 0.59-0.95) and prostate cancer (pooled SIR: 0.53, 95% CI: 0.48-0.59), compared to individuals in the general population. A higher risk of overall cancer was observed for mood, anxiety, and stress-related disorders, as well as autism and intellectual disability, in multiple studies; however, the results differed by cancer types and sex. In summary, these findings suggest an altered risk of cancer among individuals with mental illness living in Nordic countries. Further research is needed to enable the development of targeted cancer prevention strategies to reduce mental health disparities in cancer protection.

## Introduction

Cancer and mental illness constitute two major contributors to the global burden of disease. In 2020, there were 19.3 million new cancer cases and ten million cases of cancer-related death worldwide [1]. Despite advances in cancer diagnosis and treatment, and the resulting continual improvements in cancer survival, over the past decades, the health burden due to cancer is still substantial. The average years of life lost due to cancer were 15.2 in 2020 and the global economic cost for cancer is estimated at as high as $25.2 trillion from 2020 to 2050 [2]. Mental illness, including psychiatric disorders (e.g., schizophrenia, mood disorders, anxiety, and stress-related disorders) and neurodevelopmental conditions (e.g., autism, attention deficit hyperactivity disorder [ADHD], and intellectual disability), is also one of the most common causes of morbidity today. In 2019, approximately 970 million people worldwide were reported to experience mental illness, and the global number of disability-adjusted life-years due to mental illness increased from 80.8 million in 1990 to 125.3 million in 2019 [3]. In total, mental illness was estimated to cost the world economy approximately $2.5 trillion per year in poor health and reduced productivity in 2010, a cost estimated to increase to approximately $6 trillion per year by 2030 [4].

Given their high prevalence, comorbidity of cancer and mental illness is inevitable. It is well established that cancer patients have increased risks for mental illness. We and others have, for instance, demonstrated an increased risk of mental illness during the entire survivorship of cancer patients, especially immediately following cancer diagnosis [5, 6]. Although it is difficult to discern the exact reasons for this risk increment, severe psychological distress experienced during cancer diagnosis and treatment, in addition to cancer biology itself and side effects of cancer treatment, might play a role. Importantly, we have also demonstrated that comorbid mental illness before or after cancer diagnosis increases the risk of overall and cancer-specific mortality [7, 8], as well as other severe adverse outcomes (e.g., sepsis) after cancer diagnosis [9]. On the other hand, conflicting findings have been observed regarding the risk of cancer among individuals with mental illness [10]. Previous systematic reviews and meta-analyses have investigated cancer incidence among individuals with schizophrenia [11, 12], depression or anxiety [13], and anorexia nervosa [14], demonstrating high levels of heterogeneity between studies. Study results also vary by cancer type, sex, and the type of mental illness. Namely, the risk of overall cancer was shown to be slightly increased among individuals with anxiety or depression, slightly decreased among individuals with schizophrenia, and unaltered among individuals with anorexia nervosa. As a comprehensive understanding of cancer risk among individuals with mental illness is essential for effective cancer prevention strategies, we aimed to conduct an updated systematic review and meta-analysis of cancer risk among individuals with a broader range of mental illness types, including both psychiatric and neurodevelopmental conditions.

## Methods

### Search strategy and selection criteria

We conducted a systematic review and meta-analysis of the risk of cancer after the diagnosis of mental illness, according to a protocol registered on PROSPERO prior to formal initiation of the review (identifier: CRD42024593919). Due to the extensive quantity of literature published, using varying quality of data, we subsequently restricted our review to studies using nationwide Nordic register data, to ensure minimal risk of bias.

As such, studies were eligible for inclusion if they used Nordic registers to define both the exposure and outcome variables, using diagnostic codes, for the entire national population. One or more of the following types of mental illness must have been included as the exposure variable(s): schizophrenia, mood disorders (including bipolar disorder and depression), anxiety (including obsessive-compulsive disorder [OCD]) and stress-related disorders, eating disorders, and neurodevelopmental conditions (including autism, ADHD, and intellectual disability). Studies were eligible for inclusion if they used the risk of ‘any cancer’ as a combined outcome, and/or studied one or more of the following most prevalent cancer types in Europe: breast, colorectal, lung, prostate, urinary bladder, uterine, kidney, or pancreatic cancer [15]. Additionally, studies must have conducted prospective analysis of the association between the exposure (diagnosis of mental illness) and the outcome (cancer incidence) (i.e., cohort/nested case-control studies), quantified using one of the following measures of association: odds ratio (OR), hazard ratio (HR), relative risk (RR), risk ratio (RR), incidence rate ratio (IRR), standardized incidence ratio (SIR), or standardized morbidity ratio (SMR).

Studies were excluded if they focused on paediatric or elderly populations, or cancer outcomes (e.g., survival or treatment) other than incidence. Studies using other designs (i.e., cross-sectional studies, case studies, case series, randomized controlled trials [RCTs], or qualitative studies), systematic reviews, conference abstracts, and studies not published in English or subsequently translated to English were also excluded.

We searched five electronic databases (PubMed, Embase, PsycINFO, Web of Science, and Scopus) on October 24, 2024, to identify eligible studies published since database initiation. Search terms were selected after reviewing keywords in known eligible literature. The full search strategies used for each database are displayed in Supplementary Table 1.

Title and abstract screening and full text review were conducted by XX and GM, with 20% of articles independently screened by FS at each stage. Any disagreement was resolved by discussion with MMB and FF. Reference lists of all relevant systematic reviews, and all included studies, were searched for additional eligible studies. Study authors were contacted if articles contained missing data for the association measures.

### Data analysis

Data were extracted by XX, FS and MMB, using a pre-piloted data extraction sheet, and all extracted data were checked for accuracy by FF. Key study characteristics including country of study, study population, definitions of exposure and outcome variables, age, and sex were extracted.

Risk of bias was assessed by MMB and FF using the Joanna Briggs Institute (JBI) critical appraisal checklist for cohort studies [16]. This tool contains 11 questions to assess the potential risk of bias in specific study domains, each of which can be answered with ‘yes’, ‘no’, ‘unclear’, or ‘not applicable’. As certain questions did not apply to all types of study design, we calculated a total score for each study according to the number of questions for which ‘yes’ had been selected, divided by the number of applicable questions for the respective study, which was then converted into a percentage score. Based on previous literature [17], studies were classified as being at low risk of bias if they scored 70% or above, at moderate risk of bias if they scored between 50-60%, and at high risk of bias if they scored under 50%.

Random effects meta-analyses were conducted when studies from at least three countries were available for the relevant exposure (i.e., mental illness type). Where multiple studies from each country were performed using the same exposure and outcome, studies were selected for meta-analyses based on the measure of association used and the date of publication. For the main meta-analyses models we included only studies that used standardized incidence ratio (SIR), as this was the association measure used in at least one study from all included countries. We also conducted a sensitivity analyses, in which the most recent study from each country was included in each meta-analysis, regardless of the measure of association used. This allowed us to ascertain whether the observed associations persisted in the most recently conducted studies. However, bias introduced by combining different estimands with different reference groups may have reduced the validity of these pooled estimates. The maximally adjusted models in each study were included in the meta-analyses. Meta-analyses were performed using STATA version 18, and heterogeneity was measured using the I² statistic. Publication bias was assessed using the Egger’s test. For exposures not eligible for meta-analysis, a narrative synthesis was conducted.

### Role of the funding source

The funder of the study had no role in study design, data collection, data analysis, data interpretation, or writing of the report.

## Results

**Fig. 1:**
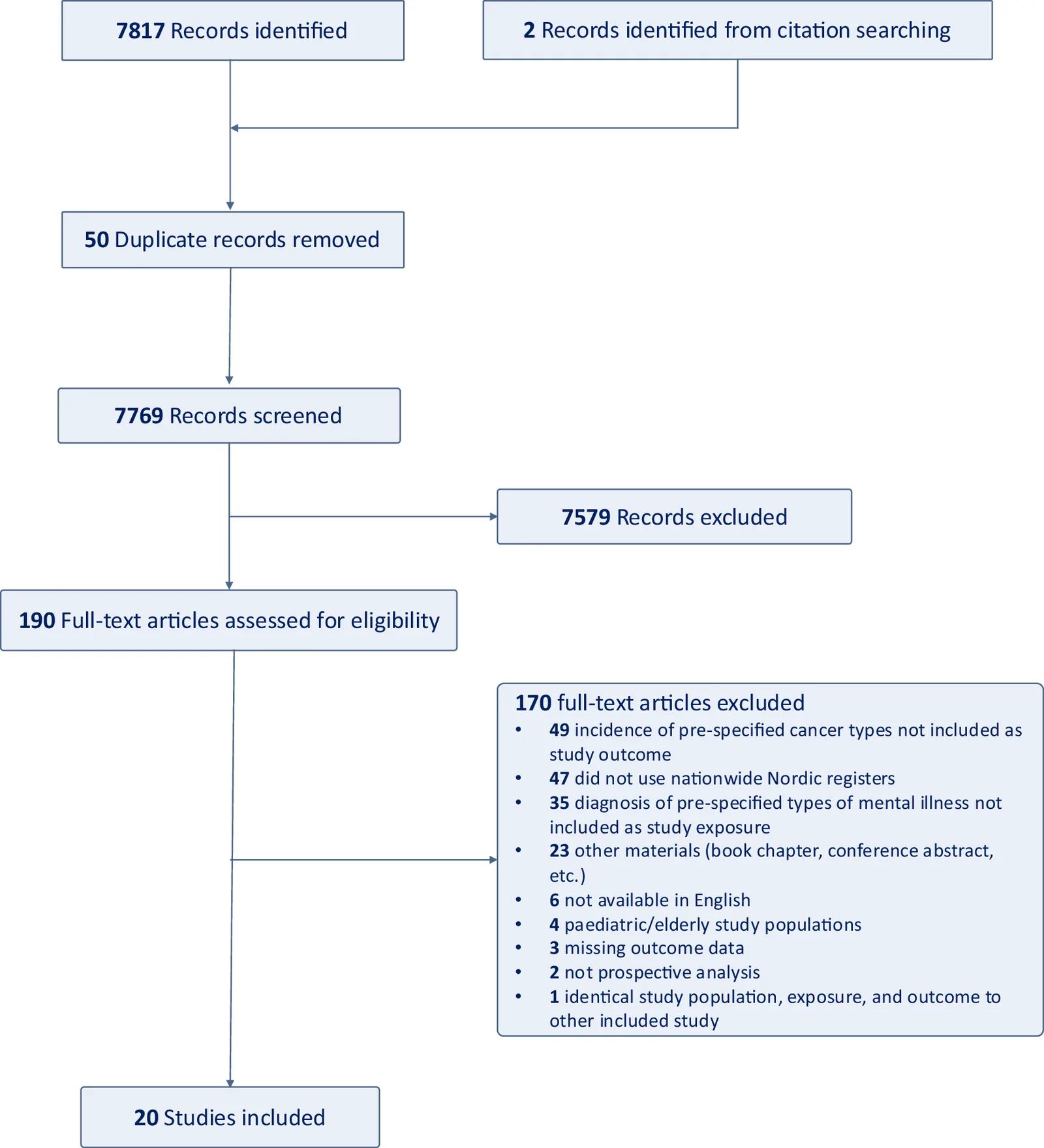
Flow diagram of study selection.

From the database searches, 7769 non-duplicate articles were identified, of which 190 full texts were reviewed for eligibility, and 20 were included in the systematic review (Fig. 1).

### Study characteristics

The key characteristics of the 20 included studies are displayed in Table 1. A total of ten studies [18–27] (50%) were from Sweden, eight studies [28–35] (40%) were from Denmark, one study [36] was from Finland, and one was a multinational Nordic study [37] using data from Sweden, Denmark, and Finland.

**Table 1:** Key characteristics of the 20 included studies.

Study	Country	Study period	Study design	Study population size	Measure of association used	Age-based eligibility criteria	Average follow-up time (years)	% female	Risk of bias
Crump et al., [18]^a^	Sweden	2003-2009	Cohort	6,587,036 individuals (including 8277 with diagnosis of schizophrenia)	HR	Aged ≥25 years	NR	42	Low
Crump et al., [19]^b^	Sweden	2003-2009	Cohort	6,097,834 (including 6618 with diagnosis of bipolar disorder)	HR	Aged ≥20 years	NR	59	Low
Dalton et al., [28]	Denmark	1969-1995	Cohort	89,491 individuals with diagnosis of mood disorder^+^	SIR	Aged ≥15 years	12.5	64	Low
Dalton et al., [29]	Denmark	1970-1997	Cohort	1,336,313 individuals (including 7541 with diagnosis of schizophrenia)	RR	Born after 1935, aged ≥25 years	9.9	100 (female-only study)	Low
Dalton et al., [30]	Denmark	1969-1995	Cohort	22,766 individuals with diagnosis of schizophrenia^+^	SIR	Aged ≥15 years	12.8	43	Low
Gradus et al., [31]	Denmark	1995-2011	Cohort	4131 individuals with diagnosis of PTSD^+^	SIR	Aged ≥16 years	7.6	60	Low
Ji et al., [20]	Sweden	1965-2008	Cohort	59,232 individuals with diagnosis of schizophrenia^+^	SIR	None	NR	46	Low
Karamanis et al., [21]	Sweden	1973-2003	Cohort	6009 indidviduals with diagnosis of anorexia nervosa^+^	SIR	Aged 10-40 years	15.2	100 (female-only study)	Low
Lichtermann et al., [36]	Finland	1971-1996	Cohort	26,996 individuals with diagnosis of schizophrenia^+^	SIR	Born between 1940-1969	16.5	42	Low
Liu et al., [22]	Sweden	1974-2016	Cohort	3,531,305 individuals (including 27,956 with diagnosis of intellectual disability)	HR	Born between 1974-2013	8.9 (median)	42	Low
Liu et al., [23]	Sweden	1987-2016	Cohort	2,354,594 individuals (including 40,334 with diagnosis of autism)	OR	Born between 1987-2013	NR	32	Low
Lu et al., [24]	Sweden	1990-2009	Nested case-control	94,626 breast cancer cases and 2,838,765 cancer-free controls	OR	Aged ≥18 years	15.2	100 (female-only study)	Low
Mellemkjaer et al., [32]	Denmark	1970-1996	Cohort	2337 individuals diagnosed with anorexia nervosa^+^	SIR	Aged 7-50 years	11.7 (females), 12.5 (males)	92	Low
Mellemkjaer et al., [37]	Sweden, Denmark & Finland	1969-2011*	Cohort	265,581 individuals (including 24,332 with diagnosis of anorexia nervosa)	IRR	Aged 10-50 years	12.6	93	Low
Michels et al., [25]	Sweden	1965-2000	Cohort	7303 individuals with diagnosis of anorexia nervosa^+^	SIR	Aged <40 years	13.3	100 (female-only study)	Low
Momen et al., [33]	Denmark	2000-2016	Cohort	5,940,299 individuals (including 698,874 with diagnosis of at least one mental illness^++^)	HR	Born between 1900-2015, aged ≥1 year	NR	50 (of entire study population)	Low
Mortensen, [34]	Denmark	1957-1984	Cohort	6152 indivdiauls with diagnosis of schizophrenia^+^	SIR	None	NR	52	Moderate
Pettersson et al., [26]	Sweden	1990-2013	Cohort	111,306 individuals with diagnosis of schizophrenia	IRR	Aged ≥15 years	12.8	53	Low
Tian et al., [27]	Sweden	1981-2016	Cohort	1,799,637 indidviduals (including 167,836 with diagnosis of stress-related disorders)	HR	Born between 1932-2011, aged ≥5 years	7.1	62	Low
Wium-Andersen et al., [35]	Denmark	1997-2018	Cohort	413,844 individuals (including 246,283 with diagnosis of mood disorder)	HR	Aged ≥18 years	9.5	51	Low

PTSD: post-traumatic stress disorder; *HR* hazard ratio, *SIR* standardized incidence ratio, *OR* odds ratio, *IRR* incidence rate ratio, *RR* relative risk, *NR* not reported

^a^ Crump C, Winkleby MA, Sundquist K, Sundquist J. Comorbidities and mortality in persons with schizophrenia: a Swedish national cohort study. *American Journal of Psychiatry*. 2013;170[3]:324-333.

^b^ Crump C, Sundquist K, Winkleby MA, Sundquist J. Comorbidities and mortality in bipolar disorder: a Swedish national cohort study. *JAMA psychiatry*. 2013;70[9].

*study period varied by country - Sweden: 1973-2010, Denmark: 1970-2011, Finland: 1969-2009.

^+^Cancer incidence compared to incidence rates in respective national population.

^++^ relevant mental illness for this review included: schizophrenia, mood disorders (including bipolar and depression), anxiety and stress-related disorders, eating disorders, and neurodevelopmental disorders (including attention deficit hyperactivity disorder [ADHD] and autism spectrum disorder [ASD]).

Average follow-up time refers to mean, unless otherwise specified. Average follow-up time and % female columns refer to exposed individuals, unless otherwise specified.

The majority of included studies (N = 19, 95%) were cohort studies, while one was a nested case-control study. Of the 19 included cohort studies, nine compared cancer incidence in exposed vs unexposed participants, using hazard ratios (HR), relative risk (RR), incidence rate ratios (IRR) or odds ratios (OR). The ten remaining cohort studies compared cancer incidence between exposed participants and the total national population of interest, using standardized incidence ratios (SIR) or incidence rate ratios (IRR). The nested case-control study compared odds of mental illness between cases of breast cancer and controls, using OR.

Age-based eligibility criteria were used in 18 (90%) of the included studies. Average follow-up time was reported in 14 studies (70%), with a mean/median ranging from 7.1 years to 16.5 years. The proportion of female participants was reported in all studies, ranging from 32% to 100%. Of the 20 included studies, 19 were classified as being at low risk of bias, and one at moderate risk of bias, according to the JBI Critical Appraisal Checklist for Cohort Studies (Supplementary Table 2). However, seven (35%) of the included studies did not clearly describe whether participants were free of the outcome at study start. For the identification of mental illness, 11 studies (55%) used data from both inpatient and outpatient care records, eight studies (40%) used inpatient care records only, and one study used data from inpatient care and disability pension records (Supplementary Table 3). The model results from each study, including the covariates adjusted for, are displayed in Supplementary Tables 4 and 5, and differed greatly between studies.

### Schizophrenia

**Fig. 2:**
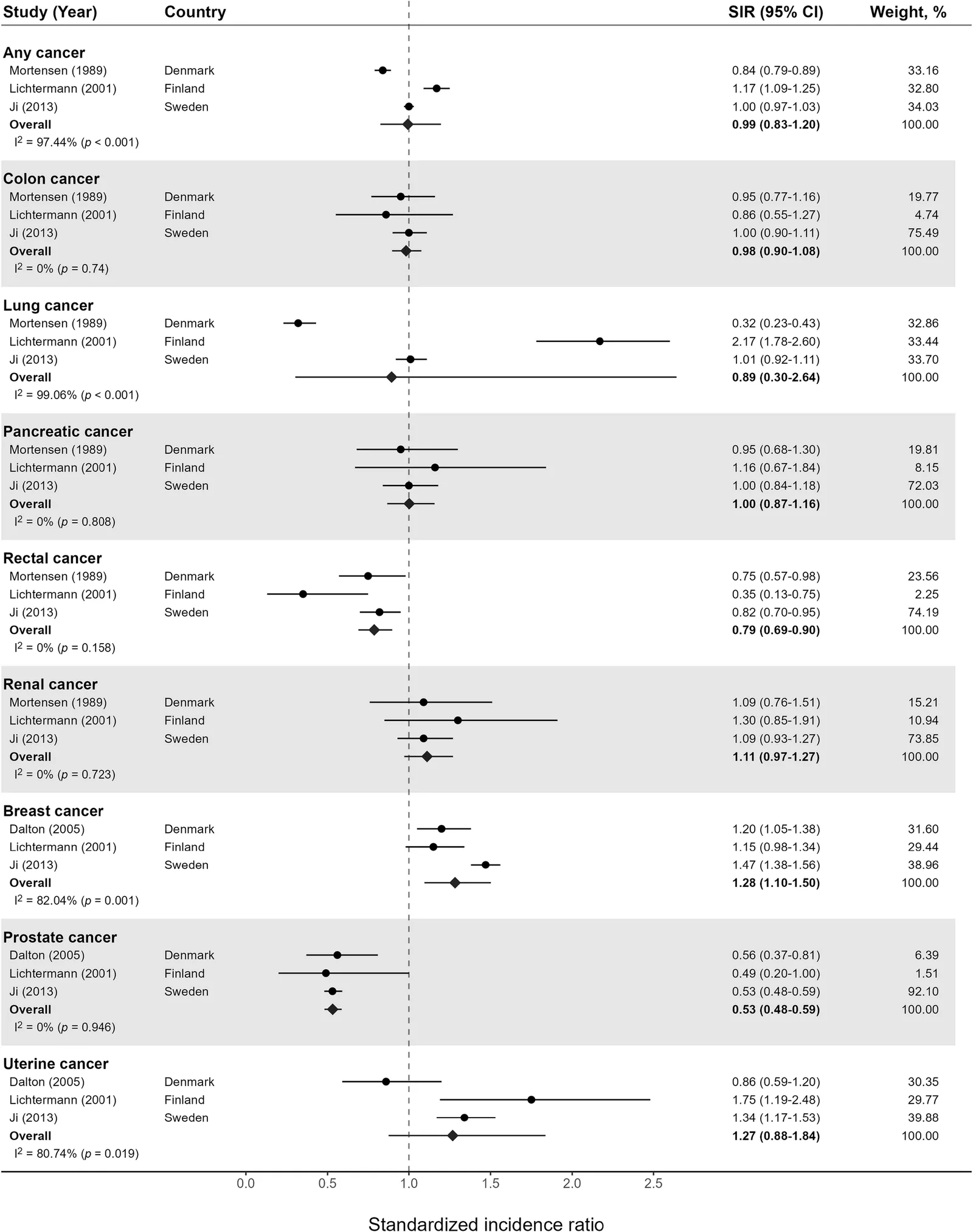
Meta-analyses of the most recent studies from each country using standardized incidence rates (SIR) to measure the association between schizophrenia diagnoses and the risk of each cancer type.

In total, nine studies from Denmark, Sweden, and Finland investigated the association between diagnoses of schizophrenia and subsequent cancer risk [18, 20, 24, 26, 29, 30, 33, 34, 36]. Meta-analyses of the most recent study from each country utilizing SIR showed no consistent association between countries, as the levels of heterogeneity between studies were high (I^2^: 97.4%, *p* < 0.001) (Fig. 2). Individuals with a diagnosis of schizophrenia appeared to have a higher risk of breast cancer (pooled SIR: 1.28, 95% CI: 1.10-1.50), and a lower risk of both rectal cancer (pooled SIR: 0.79, 95% CI: 0.69-0.90) and prostate cancer (pooled SIR: 0.53, 95% CI: 0.48-0.59). However, the levels of heterogeneity remained high (e.g., I²: 82% for breast cancer).

**Fig. 3:**
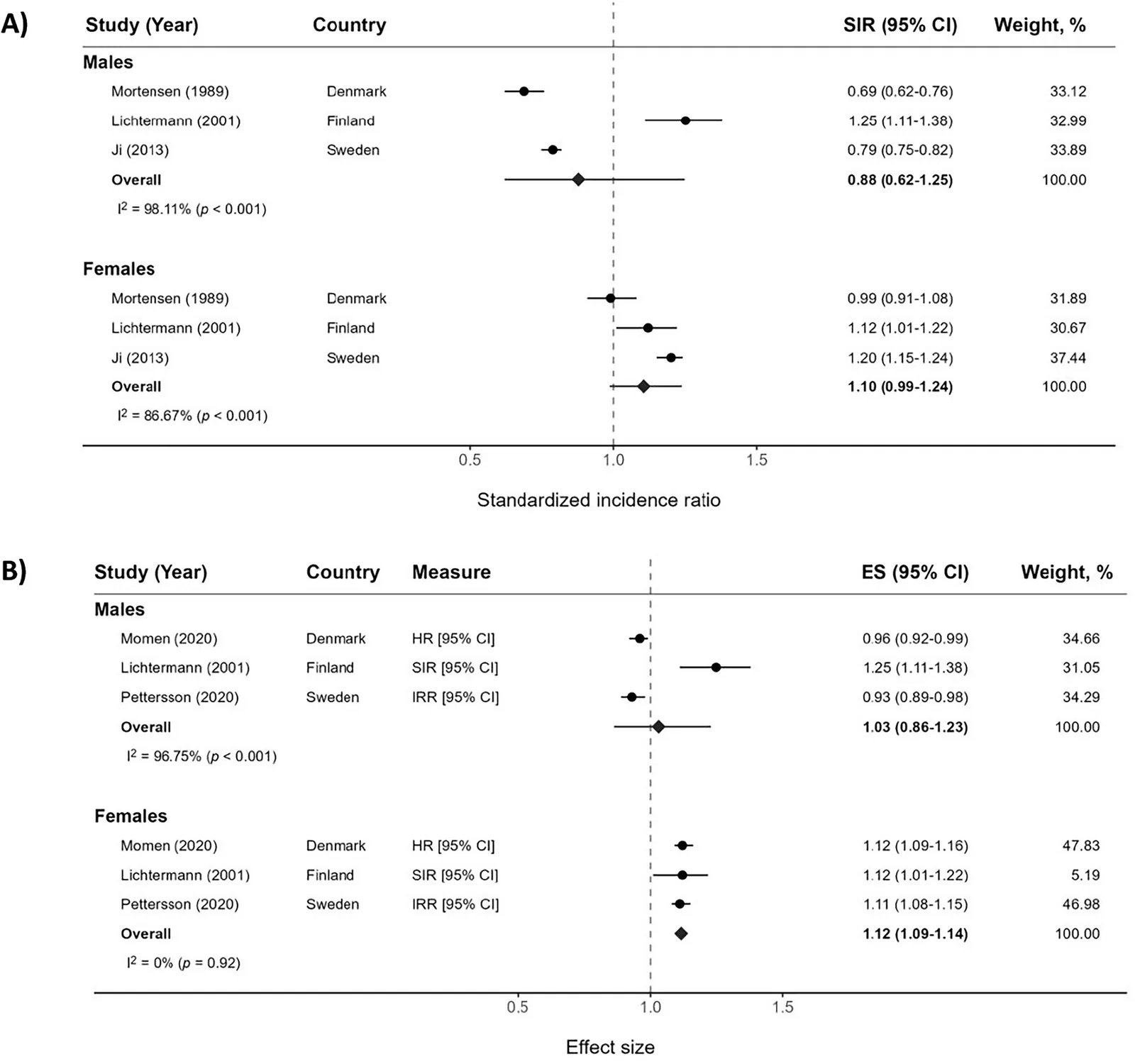
Sex-stratified meta-analyses of most recent studies from each country investigating the association between schizophrenia diagnoses and the risk of any cancer. (**A**) Standardized incidence rates (SIR) used. (**B**) Regardless of measure of association used. Pooled estimates combine standardized incidence ratios (SIR), odds ratios (OR), hazard ratios (HR), and incidence rate ratios (IRR).

Similar associations were observed in the sensitivity analyses, in which the most recent study from each country was included in each meta-analysis, regardless of the measures of association used, i.e., combining HR, SIR, IRR, and OR to calculate the pooled estimates (Supplementary Figure 1). Sex-stratified meta-analyses showed a difference in the association between diagnoses of schizophrenia and risk of any cancer in men, compared to women; however, none of the results from the sex-stratified meta-analyses were statistically significant, and levels of heterogeneity between studies were high (Fig. 3). No evidence was found for publication bias in the meta-analyses (Supplementary Table 6).

### Mood disorders

Of the included studies, four studies conducted in Denmark and Sweden investigated the associations between diagnoses of mood disorders and subsequent cancer risk. One study [33] showed a slightly higher risk of any cancer among individuals with a diagnosis of any mood disorder (HR: 1.03, 95% CI: 1.02-1.05), while another study [19] observed no association between bipolar disorder and the risk of any cancer in either males or females. A further study [28] categorized mood disorders into bipolar disorder and other affective disorders, finding that individuals with other depressive disorders had a higher risk of any cancer (SIR: 1.14, 95% CI: 1.10-1.17), lung cancer (SIR: 1.59, 95% CI: 1.47-1.72), colon cancer (SIR: 1.16, 95% CI: 1.03-1.30), pancreatic cancer (SIR: 1.52, 95% CI: 1.27-1.79), and urinary bladder cancer (SIR: 1.23, 95% CI: 1.07-1.41), while individuals with bipolar disorder had a lower risk of rectal cancer (SIR: 0.79, 95% CI: 0.68-0.92). The remaining study [35] categorized mood disorders into bipolar disorder, major depressive disorder, and other affective disorders, finding that individuals with major depressive disorder had a higher risk of lung cancer (HR: 1.12, 95% CI: 1.04-1.20), and a slightly lower risk of prostate cancer (HR: 0.91, 95% CI: 0.83-0.99).

### Anxiety and stress-related disorders

In total, three studies from Denmark and Sweden analyzed associations between diagnoses of anxiety or stress-related disorders and subsequent cancer risk. One study [33] used a broad definition of anxiety and stress-related disorders, finding a higher risk of any cancer among individuals with diagnoses of such disorders in the overall study population (HR: 1.13, 95% CI: 1.11-1.15), and among males and females separately. Another study [27] used stress-related disorders as the exposure, finding a higher risk of any cancer (HR: 1.03, 95% CI: 1.01-1.06), lung cancer (HR: 1.25, 95% CI: 1.15-1.37) and pancreatic cancer (HR: 1.50, 95% CI: 1.28-1.75), but a lower risk of prostate cancer (HR: 0.89, 95% CI: 0.82-0.96) among individuals with such diagnoses. The remaining study [31], which focused only on diagnoses of post-traumatic stress disorder (PTSD), found no association with the risk of any cancer or specific cancer types (including breast and colon cancers).

### Eating disorders

A total of five studies have investigated associations between diagnoses of eating disorders and subsequent cancer risk. One study [33] defined eating disorders as diagnoses of anorexia nervosa or bulimia nervosa, finding that individuals with such diagnoses had a higher risk of any cancer (HR: 1.26, 95% CI: 1.14-1.39). However, sex-stratified analysis showed that the association was only observed in females. The remaining four studies [21, 25, 32, 37] used diagnoses of anorexia nervosa as the study exposure, finding no association between such diagnoses and the risk of any cancer. However, another study [37] did observe a higher risk of lung cancer (IRR: 1.60, 95% CI: 1.10-2.40) among females diagnosed with anorexia nervosa, whereas two additional studies [25, 37] demonstrated a lower risk of breast cancer (e.g., IRR: 0.60, 95% CI: 0.50-0.80) among females with such diagnoses.

### Neurodevelopmental conditions

One study [23], conducted in Sweden, investigated the association between diagnoses of autism and subsequent cancer risk, finding a higher risk of any cancer among individuals with such diagnoses (OR: 1.30, 95% CI: 1.20-1.50). Two studies, conducted in Sweden and Denmark, analyzed associations between diagnoses of intellectual disability and subsequent risk of cancer. One study [22] showed a higher risk of any cancer among individuals with intellectual disability (HR: 1.57, 95% CI: 1.35-1.82). Although the other study [33] found no association between such diagnoses and risk of any cancer in the total study population. Sex-stratified analyses revealed contrasting trends by sex, namely a lower risk of any cancer among men with intellectual disability (HR: 0.81, 95% CI: 0.73-0.90) but a higher risk of any cancer for women with such diagnoses (HR: 1.15, 95% CI: 1.04-1.27).

## Discussion

This systematic review of 20 prospective Nordic register-based studies indicates an altered risk of cancer among individuals with mental illness, compared to the general population. However, findings varied greatly by the type of mental illness and cancer type. Sex variations were also observed. Although no association between schizophrenia and overall cancer risk was found in the meta-analyses, our site-specific cancer findings align with previous systematic reviews, showing a higher risk of breast cancer, and a lower risk of prostate and rectal cancer among individuals with schizophrenia [11, 12]. Specific types of mood, anxiety, and stress-related disorders were associated with an increased risk of overall cancer in some studies, supporting results from a previous systematic review of cancer risk among individuals with depression or anxiety [13]. Individuals with eating disorders did not have an altered risk of overall cancer in the majority of the included studies; however, a reduced risk of breast cancer was observed among women, aligning with a previous systematic review of cancer risk among individuals with anorexia nervosa [14]. Furthermore, findings from the included studies suggested a higher risk of overall cancer among individuals with autism or intellectual disability. There is currently limited knowledge on the risk of cancer among individuals with other neurodevelopmental conditions, including ADHD, as well as specific types of anxiety disorders (e.g., obsessive-compulsive disorder [OCD]).

An altered risk of cancer among individuals with mental illness is plausible and potentially attributable to a multitude of factors [10]. Firstly, behavioural and lifestyle factors (e.g., smoking, excessive alcohol use, obesity, poor diet, and physical inactivity), which are more prevalent among individuals with mental illness [38], are also established cancer risk factors [39]. Poorer socioeconomic circumstances (e.g., lower education and income, suboptimal social integration, and greater unemployment) among individuals with mental illness may also exacerbate cancer risk [40]. Detection biases related to mental illness may also contribute to the differing risks of various cancer types observed among individuals with different types of mental illness. Accordingly, mental illness is often accompanied by reduced access to, and quality of, health care [41], potentially making it difficult for individuals with mental illness to be diagnosed with, and treated for, precancerous conditions in a timely manner. As such, individuals with mental illness do not appear to benefit equally from existing preventive strategies for cancer, as evidenced by persistently lower cancer screening utilization [42]. Lower screening participation among individuals with mental illness could result in the reduced detection of cancer, which might partially explain the lower-than-expected risk of rectal cancer among individuals with schizophrenia and of prostate cancer among individuals with schizophrenia, mood disorders, or anxiety disorders. Previous research has indeed indicated that individuals with schizophrenia have lower participation in fecal occult blood testing and even lower completion rates of follow-up colonoscopy [43, 44]. Conversely, the higher-than-expected risk of certain cancer types among individuals with mood or anxiety disorders might be partly attributed to heightened somatic worry and health concerns among these patients, which may increase their healthcare-seeking behaviours and the subsequent likelihood of cancer detection. Further, treatment for mental illness, such as antipsychotic drugs, have been shown to affect cancer risk through weight gain and obesity, hormonal changes [45], and hyperprolactinemia, which can increase the risk of breast cancer [46]. However, protective effects of antipsychotic drugs have also been observed for certain cancers such as prostate cancer [12]. Additionally, as many mental illnesses, especially the severe types, are associated with an increased risk of chronic somatic diseases, competing risk due to non-cancer-related death might also modulate the risk of cancer diagnosis, which could partially explain the inverse association observed between schizophrenia and prostate cancer. Nevertheless, further research is needed to identify which individuals with mental illness are at particularly high risk of cancer and the reasons contributing to this elevated risk, in order to design effective cancer prevention strategies.

Shared genetic liability could also contribute to an increased or decreased risk of cancer among individuals with mental illness [47]. Genome-wide association studies (GWAS) have revealed shared genetic mechanisms underlying multiple diseases, including mental disorders and cancer [24, 48–52]. Like many other diseases, evidence suggests that mental illness and cancer are not caused by genetic or environmental factors alone, but rather by a complex interplay between nature and nurture (or ‘gene-environment interactions’) [53, 54]. Indeed, a large number of studies have examined potential interactions between environmental cancer risk factors and individual genetic predispositions to cancer measured by family history and the presence of specific genes [54]. However, few studies have assessed the effect of the interaction between mental illness and individual genetic predisposition to cancer on subsequent cancer incidence. Interestingly, the risk increment of cancer noted among children with autism has been observed to decline with increasing heritability of cancer type, suggesting that autism may play a greater role in less heritable cancer types [23]. These findings highlight the potential of future family-design studies to disentangle the role of environment and genetics in the comorbidity between mental illness and cancer.

There are many strengths of this review. Firstly, the broad range of types of mental illness included allows for a comprehensive understanding of the associations between mental illness and cancer risk. Additionally, the inclusion of only Nordic register studies in the review increases the validity of our conclusions, as these studies included the entire national population with prospectively and independently collected information on mental illness and cancer, therefore greatly alleviating the risk of selection and information biases. The high quality of the included studies is highlighted in our risk of bias appraisal, whereby the vast majority of studies were classified as being at low risk of bias. Furthermore, we used a robust search strategy to search five research databases, in addition to searching reference lists of eligible studies and relevant systematic reviews, which allowed us to identify all eligible studies, thereby increasing the robustness of our conclusions.

However, some limitations must be noted. Firstly, only studies published in English were included in the review, potentially reducing the generalizability of our conclusions. Our review was also limited by the available data set. Although the use of Nordic register studies diminished the risk of biases, the lack of adjustment for key confounders such as body mass index (BMI) and smoking, due to the lack of such data in the registers, increases the risk of residual confounding. Furthermore, as weight gain is a common side effect of many psychotropic medications, BMI could act as mediator in the associations between mental illness and cancer risk, which was rarely accounted for in the included studies. Additionally, as several of the included studies did not specify whether participants were free of the outcome (i.e., cancer) when the exposure was assessed, temporality issues could have reduced the validity of the observed associations. Therefore, future studies should ensure that cancer has not occurred before the onset of mental illness, for example by using a ‘wash out period’ after the first diagnosis of mental illness to ensure that only incident cancer cases occurring after the exposure are identified. Between-study variations in model adjustments and the ICD definitions used for mental illness also increased heterogeneity and made comparisons between studies difficult. Further, for most of the mental illness types, studies had only been conducted in Sweden and Denmark, meaning that we could not perform meta-analyses for these exposures. However, the narrative syntheses allowed us to describe and compare the findings from the included studies. Finally, caution is needed when generalizing the study findings to other settings, due to the unique healthcare and social welfare systems in the Nordic countries.

In conclusion, this systematic review demonstrates an altered risk of cancer among individuals with certain types of mental illness in the Nordic countries; however, the results vary greatly by cancer type and sex. We call for further focused research to better understand the factors underlying the high comorbidity between mental illness and cancer, with the aim to provide targeted prevention and reduce cancer disparities among people with mental illness.

## Supplementary information

Supplementary Material

## Data Availability

STATA code used to generate meta-analyses can be made available upon request.
