# Supplementary material for: Cancer risk among individuals with mental illness – a systematic review and meta-analysis of nordic register-based prospective studies
Source: Transl Psychiatry. 2026 Aug 1;16:595. doi: 10.1038/s41398-026-04351-5 (PMC13719996; doi:10.1038/s41398-026-04351-5)
Supplement: Supplementary file 1 [file 41398_2026_4351_MOESM1_ESM.docx]

**Supplementary Material**

**Cancer risk among individuals with mental illness – A systematic review and meta-analysis of Nordic register-based prospective studies**

Xue Xiao, Fatemeh Sadeghi, Gillian Murphy, Zhe Zhang, Ole A. Andreassen, Unnur Valdimarsdóttir, Fang Fang, Mary M Barker

Table of Contents

[Supplementary Table 1: Database search strategies	3](#_Toc219104615)

[Supplementary Table 2: Risk of bias of the included studies, according to the Joanna Briggs Institute (JBI) critical appraisal checklist for cohort studies	5](#_Toc219104616)

[Supplementary Table 3: Definitions of types of mental illness used in the 20 included studies	7](#_Toc219104617)

[Supplementary Table 4: Results from models including total study population, by type of mental illness and cancer	9](#_Toc219104618)

[Supplementary Table 5: Sex-stratified model results by type of mental illness and cancer	13](#_Toc219104619)

[Supplementary Table 6: Results from Egger’s test for publication bias in the meta-analyses	17](#_Toc219104620)

[Supplementary Figure 1: Meta-analyses of the most recent studies from each country investigating the association between schizophrenia diagnoses and the risk of each cancer type, regardless of measure of association used	18](#_Toc219104621)

[PRISMA Checklist	19](#_Toc219104622)

# **Supplementary Table 1:** Database search strategies

| Database | Number of records identified | Search Strategy |
| --- | --- | --- |
| EMBASE | 3461 | ('mental disease'/exp OR 'anxiety'/exp OR 'depression'/exp OR 'schizophrenia'/exp OR 'eating disorder'/exp OR 'autism'/exp OR 'mental disease'/exp/mj/dm_ep OR 'mental disease'/exp/mj OR 'anxiety'/exp/mj OR 'depression'/exp/mj OR 'schizophrenia'/exp/mj OR 'eating disorder'/exp/mj OR 'autism'/exp/mj OR 'autism'/exp/mj OR 'attention deficit hyperactivity disorder'/exp/mj OR ('mental*':ti OR 'psychiatric*':ti OR 'psychologic*':ti OR 'neuropsych*':ti OR 'mood disorder*':ti OR 'affective disorder*':ti OR 'depressi*':ti OR 'anxi*':ti OR 'phobi*':ti OR 'obsessive compulsive*':ti OR 'ocd':ti OR 'panic*':ti OR 'stress*':ti OR 'post traumatic*':ti OR 'posttraumatic*':ti OR 'ptsd':ti OR 'bipolar*':ti OR 'schizophren*':ti OR 'autis*':ti OR 'autism spectrum*':ti OR 'adhd':ti OR 'attention deficit*':ti OR 'eating disorder*':ti OR 'anorexi*':ti OR 'bulimi*':ti OR 'schizoaffect*':ti OR 'psychotic*':ti OR 'psychosis*':ti)) AND ('neoplasm'/exp OR 'neoplasm*':ti OR 'cancer*':ti OR 'tumor*':ti OR 'tumour*':ti OR 'malignan*':ti) AND ('Sweden'/exp OR 'Norway'/exp OR 'Finland'/exp OR 'Denmark'/exp OR 'Iceland'/exp OR 'Scandinavia'/exp OR 'sweden':ti,ab,kw OR 'swedish':ti,ab,kw OR 'swed*':ti,ab,kw OR 'norway':ti,ab,kw OR 'norwegian*':ti,ab,kw OR 'finland':ti,ab,kw OR 'finnish':ti,ab,kw OR 'finn*':ti,ab,kw OR 'denmark':ti,ab,kw OR 'danish':ti,ab,kw OR 'dane*':ti,ab,kw OR 'iceland':ti,ab,kw OR 'iceland*':ti,ab,kw OR 'nordic':ti,ab,kw OR 'scandinavia*':ti,ab,kw OR 'scandinavian':ti,ab,kw) |
| PubMed | 1197 | ("mental disorders"[MeSH Terms] OR "anxiety"[MeSH Terms] OR "depression"[MeSH Terms] OR "schizophrenia"[MeSH Terms] OR "feeding and eating disorders"[MeSH Terms] OR "autistic disorder"[MeSH Terms] OR "mental disorders/epidemiology"[MeSH Major Topic] OR "mental disorders"[MeSH Major Topic] OR "anxiety"[MeSH Major Topic] OR "depression"[MeSH Major Topic] OR "schizophrenia"[MeSH Major Topic] OR "feeding and eating disorders"[MeSH Major Topic] OR "autistic disorder"[MeSH Major Topic] OR "autism spectrum disorder"[MeSH Major Topic] OR "attention deficit disorder with hyperactivity"[MeSH Major Topic] OR ("mental*"[Title] OR "psychiatric*"[Title] OR "psychologic*"[Title] OR "neuropsych*"[Title] OR "mood disorder*"[Title] OR "affective disorder*"[Title] OR "depressi*"[Title] OR "anxi*"[Title] OR "phobi*"[Title] OR "obsessive compulsive*"[Title] OR "OCD"[Title] OR "panic*"[Title] OR "stress*"[Title] OR "post traumatic*"[Title] OR "posttraumatic*"[Title] OR "PTSD"[Title] OR "bipolar*"[Title] OR "schizophren*"[Title] OR "autis*"[Title] OR "autism spectrum*"[Title] OR "ADHD"[Title] OR "attention deficit*"[Title] OR "eating disorder*"[Title] OR "anorexi*"[Title] OR "bulimi*"[Title] OR "schizoaffect*"[Title] OR "psychotic*"[Title] OR "psychosis*"[Title])) AND ("neoplasms"[MeSH Terms] OR "neoplasm*"[Title] OR "cancer*"[Title] OR "tumor*"[Title] OR "tumour*"[Title] OR "malignan*"[Title]) AND ("sweden"[MeSH Terms] OR "norway"[MeSH Terms] OR "finland"[MeSH Terms] OR "denmark"[MeSH Terms] OR "iceland"[MeSH Terms] OR "scandinavian and nordic countries"[MeSH Terms] OR "sweden"[Title/Abstract] OR "swedish"[Title/Abstract] OR "swed*"[Title/Abstract] OR "norway"[Title/Abstract] OR "norwegian*"[Title/Abstract] OR "finland"[Title/Abstract] OR "finnish"[Title/Abstract] OR "finn*"[Title/Abstract] OR "denmark"[Title/Abstract] OR "danish"[Title/Abstract] OR "dane*"[Title/Abstract] OR "iceland"[Title/Abstract] OR "iceland*"[Title/Abstract] OR "nordic"[Title/Abstract] OR "scandinavia*"[Title/Abstract] OR "scandinavian"[Title/Abstract]) |
| PSYCINFO | 2292 | ( mental health or mental illness or mental disorder or psychiatric illness ) OR ( psychiatric disorders or mental disorder or mental illness or psychiatric disorder ) OR ( anxiety disorders or anxiety or generalized anxiety disorder ) OR ( depression or depressive disorder or depressive symptoms or major depressive disorder ) OR ( stress disorders, post-traumatic or ptsd, or trauma ) OR ( bipolar disorder or bipolar i or bipolar ii or manic depression or bipolar affective disorder or bipolar depression ) OR ( schizophrenia or psychosis or psychoses or psychotic disorder or schizophrenic disorder ) OR ( autism or asd or autism spectrum disorder or asperger's or asperger's syndrome or autistic disorder or aspergers ) OR ( eating disorders or anorexia or bulimia or disordered eating or binge eating disorder ) OR ( adhd or attention deficit hyperactivity disorder or attention deficit-hyperactivity disorder ) OR ( ocd or obsessive-compulsive disorder or obsessive compulsive disorder ) OR ( neuropsychiatric disorders or neuropsychiatric disability or adhd or asd ) AND ( tumor or cancer or neoplasm or carcinoma ) OR malignant neoplasm OR ( malignant tumor or cancer or malignancy ) AND ( scandinavia, or norway, or sweden, or denmark, or scandinavian, or norwegian, or swedish, or danish ) OR ( nordic countries or finland or sweden or denmark or norway or iceland ) OR finnish |
| Scopus | 2063 | (TITLE-ABS-KEY ("mental disorder*") OR TITLE-ABS-KEY ("mental illness*") OR TITLE-ABS-KEY ("anxiety") OR TITLE-ABS-KEY ("depression") OR TITLE-ABS-KEY ("major depressive disorder") OR TITLE-ABS-KEY ("schizophrenia") OR TITLE-ABS-KEY ("bipolar") OR TITLE-ABS-KEY ("feeding and eating disorder") OR TITLE-ABS-KEY ("anorexia") OR TITLE-ABS-KEY ("autistic disorder") OR TITLE-ABS-KEY ("autism spectrum disorder*") OR TITLE-ABS-KEY ("psychiatric disorder*") OR TITLE-ABS-KEY ("psychosis") OR TITLE-ABS-KEY ("neurodevelopmental disorder*") OR TITLE-ABS-KEY ("ADHD") OR TITLE-ABS-KEY ("attention deficit hyperactivity disorder") OR TITLE-ABS-KEY ("attention deficit hyperactivity disorder adhd") OR TITLE-ABS-KEY ("intellectual disability") OR TITLE-ABS-KEY ("intellectual disabilities") OR TITLE-ABS-KEY ("obsessive-compulsive disorder") OR TITLE ("mental*") OR TITLE ("psychiatric*") OR TITLE ("psychologic") OR TITLE ("neuropsych") OR TITLE ("mood disorder*") OR TITLE ("affective disorder*") OR TITLE ("depressi") OR TITLE ("anxi*") OR TITLE ("phobi*") OR TITLE ("obsessive compulsive*") OR TITLE ("OCD") OR TITLE ("panic*") OR TITLE ("stress*") OR TITLE ("post traumatic*") OR TITLE ("posttraumatic*") OR TITLE ("PTSD") OR TITLE ("schizophren*") OR TITLE ("autis*") OR TITLE ("autism spectrum*") OR TITLE ("ADHD") OR TITLE ("attention deficit*") OR TITLE ("eating disorder*") OR TITLE ("anorexi*") OR TITLE ("bulimi*") OR TITLE ("schizoaffect*") OR TITLE ("psychotic*") OR TITLE ("psychosis*")) AND (TITLE-ABS-KEY ("neoplasm*") OR TITLE-ABS-KEY ("cancer*") OR TITLE ("tumor*") OR TITLE ("tumour*") OR TITLE ("malignan*")) AND (TITLE-ABS-KEY ("sweden") OR TITLE-ABS-KEY ("swedish") OR TITLE-ABS-KEY ("swed") OR TITLE-ABS-KEY ("norway") OR TITLE-ABS-KEY ("norwegian*") OR TITLE-ABS-KEY ("finland") OR TITLE-ABS-KEY ("finnish") OR TITLE-ABS-KEY ("finn*") OR TITLE-ABS-KEY ("denmark") OR TITLE-ABS-KEY ("danish") OR TITLE-ABS-KEY ("dane*") OR TITLE-ABS-KEY ("iceland") OR TITLE-ABS-KEY ("iceland*") OR TITLE-ABS-KEY ("scandinavian and nordic countries") OR TITLE-ABS-KEY ("nordic") OR TITLE-ABS-KEY ("scandinavia*") OR TITLE-ABS-KEY ("Scandinavian")) |
| Web of Science | 2881 | (TS=("mental disorder*" OR anxiety OR depression OR schizophrenia OR "feeding and eating disorder*" OR autism OR "autism spectrum disorder*" OR ADHD OR "attention deficit disorder with hyperactivity" OR mental* OR psychiatric* OR psychologic* OR neuropsych* OR "mood disorder*" OR "affective disorder*" OR depressi* OR anxi* OR phobi* OR "obsessive compulsive*" OR OCD OR panic* OR stress* OR "post traumatic*" OR posttraumatic* OR PTSD OR bipolar* OR schizophren* OR autis* OR "ADHD" OR "attention deficit*" OR "eating disorder*" OR anorexi* OR bulimi* OR schizoaffect* OR psychotic* OR psychosis*)) AND (TS=(cancer OR neoplasm* OR tumor OR tumour OR malignan*)) AND (TS=(sweden OR norway OR finland OR denmark OR iceland OR scandinavia* OR nordic OR swed* OR finn* OR norwegian* OR danish OR dane*)) |

# **Supplementary Table 2:** Risk of bias of the included studies, according to the Joanna Briggs Institute (JBI) critical appraisal checklist for cohort studies

| **Study** | **Q1** | **Q2** | **Q3** | **Q4** | **Q5** | **Q6** | **Q7** | **Q8** | **Q9** | **Q10** | **Q11** | **Score (%)** | **Overall risk of bias** |
| --- | --- | --- | --- | --- | --- | --- | --- | --- | --- | --- | --- | --- | --- |
| Crump et al., 2013^a^ | ✓ | ✓ | ✓ | ✓ | ✓ | ? | ? | X | ✓ | NA | ✓ | 70 | Low |
| Crump et al., 2013^b^ | ✓ | ✓ | ✓ | ✓ | ✓ | ? | ? | X | ✓ | NA | ✓ | 70 | Low |
| Dalton et al., 2002 | ✓ | NA | ✓ | ✓ | ✓ | ✓ | ✓ | ✓ | ✓ | NA | ✓ | 100 | Low |
| Dalton et al., 2003 | ✓ | ✓ | ✓ | ✓ | ✓ | ✓ | ✓ | ✓ | ✓ | NA | ✓ | 100 | Low |
| Dalton et al., 2005 | ✓ | NA | ✓ | ✓ | ✓ | ✓ | ✓ | ✓ | ✓ | NA | ✓ | 100 | Low |
| Gradus et al., 2015 | ✓ | NA | ✓ | ✓ | ✓ | ✓ | ✓ | ✓ | ✓ | NA | ✓ | 100 | Low |
| Ji et al., 2013 | ✓ | NA | ✓ | ✓ | ✓ | ? | ✓ | ✓ | ✓ | NA | ✓ | 88.9 | Low |
| Karamanis et al., 2014 | ✓ | NA | ✓ | ✓ | ✓ | ✓ | ✓ | ✓ | ✓ | NA | ✓ | 100 | Low |
| Lichtermann et al., 2001 | ✓ | NA | ✓ | ✓ | ? | ? | ✓ | ✓ | ✓ | NA | ✓ | 77.8 | Low |
| Liu et al., 2021 | ✓ | ✓ | ✓ | ✓ | ✓ | ✓ | ✓ | ✓ | ✓ | NA | ✓ | 100 | Low |
| Liu et al., 2022 | ✓ | ✓ | ✓ | ✓ | ✓ | ✓ | ✓ | ✓ | ✓ | NA | ✓ | 100 | Low |
| Lu et al., 2020 | ✓ | ✓ | ✓ | ✓ | ✓ | ✓ | ✓ | ✓ | ✓ | NA | ✓ | 100 | Low |
| Mellemkjaer et al., 2001 | ✓ | NA | ✓ | ✓ | ✓ | ? | ✓ | ✓ | ✓ | NA | ✓ | 88.9 | Low |
| Mellemkjaer et al., 2015 | ✓ | ✓ | ✓ | ✓ | ✓ | ✓ | ✓ | ✓ | ✓ | NA | ✓ | 100 | Low |
| Michels et al., 2004 | ✓ | NA | ✓ | ✓ | ✓ | ✓ | ✓ | ✓ | ✓ | NA | ✓ | 100 | Low |
| Momen et al., 2020 | ✓ | ✓ | ✓ | ✓ | ✓ | ? | ? | ✓ | ✓ | NA | ✓ | 80 | Low |
| Mortensen, 1989 | ✓ | NA | ? | ✓ | ? | ? | ✓ | ✓ | ✓ | NA | ✓ | 66.7 | Moderate |
| Pettersson et al., 2020 | ✓ | NA | ✓ | ✓ | ✓ | ✓ | ✓ | ✓ | ✓ | NA | ✓ | 100 | Low |
| Tian et al., 2022 | ✓ | ✓ | ✓ | ✓ | ✓ | ✓ | ✓ | ✓ | ✓ | NA | ✓ | 100 | Low |
| Wium-Andersen et al., 2021 | ✓ | ✓ | ✓ | ✓ | ✓ | ✓ | ? | ✓ | ✓ | NA | ✓ | 90 | Low |

✓=’yes’, X=’no’, ?=’unclear’, NA=not applicable

^a^ Crump C, Winkleby MA, Sundquist K, Sundquist J. Comorbidities and mortality in persons with schizophrenia: a Swedish national cohort study. *American Journal of Psychiatry*. 2013;170(3):324-333.

^b^ Crump C, Sundquist K, Winkleby MA, Sundquist J. Comorbidities and mortality in bipolar disorder: a Swedish national cohort study. *JAMA psychiatry*. 2013;70(9).

Questions included in checklist:

1. Were the two groups similar and recruited from the same population?
2. Were the exposures measured similarly to assign people to both exposed and unexposed groups?
3. Was the exposure measured in a valid and reliable way?
4. Were confounding factors identified?
5. Were strategies to deal with confounding factors stated?
6. Were the groups/participants free of the outcome at the start of the study (or at the moment of exposure)?
7. Were the outcomes measured in a valid and reliable way?
8. Was the follow up time reported and sufficient to be long enough for outcomes to occur?
9. Was follow up complete, and if not, were the reasons to loss to follow up described and explored?
10. Were strategies to address incomplete follow up utilized?
11. Was appropriate statistical analysis used?

# **Supplementary Table 3:** Definitions of types of mental illness used in the 20 included studies

| **Study** | **Type(s) of mental illness studied** | **Definition of mental illness** | **Register(s) used to identify mental illness (inpatient/outpatient)** |
| --- | --- | --- | --- |
| Crump et al., 2013^a^ | Schizophrenia | ICD-10: F20 | Swedish Hospital Registry (inpatient) and Swedish Outpatient Registry |
| Crump et al., 2013^b^ | Bipolar disorder | ICD-10: F31 | Swedish Hospital Registry (inpatient) and Swedish Outpatient Registry |
| Dalton et al., 2002 | Mood disorders | Bipolar disorder: ICD-8: 296.19, 296.39, 298.19, 296.09, 296.29, 296.89, 296.99  Other depressive disorders: ICD-8: 298.09, 300.49, 301.19 | Danish Psychiatric Central Register (inpatient) |
| Dalton et al., 2003 | Schizophrenia | ICD-10: F20, F25; ICD-8: 295 | Danish Psychiatric Central Register (inpatient) |
| Dalton et al., 2005 | Schizophrenia | ICD-8: 295 | Danish Psychiatric Central Register (inpatient) |
| Gradus et al., 2015 | Post-traumatic stress disorder (PTSD) | ICD-10: F43.1 | Danish Psychiatric Central Register & Danish National Registry of Patients (inpatient & outpatient) |
| Ji et al., 2013 | Schizophrenia | ICD-10: F20; ICD-9 and ICD-8: 295; ICD-7: 300 | Swedish Hospital Discharge Register (inpatient & outpatient) |
| Karamanis et al., 2014 | Anorexia nervosa | ICD-10: F50.0, F50.1; ICD-9: 307B; ICD-8: 306.50 | Swedish Hospital Discharge Register (inpatient) |
| Lichtermann et al., 2001 | Schizophrenia | ICD-9 and ICD-8: 295 | National Hospital Discharge (inpatient) and Disability Pension Registers |
| Liu et al., 2021 | Intellectual disability | ICD-10: F70-73, F78, F79; ICD-9: 317, 318, 318A, 318B, 318C; ICD-8: 310-315 | Swedish National Patient Register (inpatient & outpatient) |
| Liu et al., 2022 | Autism | ICD-10: F84.0, F84.5, F84.8, F84.9; ICD-9: 299A, 299B, 299X | Swedish National Patient Register (inpatient & outpatient) |
| Lu et al., 2020 | Schizophrenia | ICD-10: F20; ICD-9 and ICD-8: 295 | Swedish National Patient Register (inpatient & outpatient) |
| Mellemkjaer et al., 2001 | Anorexia nervosa | ICD-8: 306.50 | Psychiatric Case Register and National Registry of Patients (inpatient) |
| Mellemkjaer et al., 2015 | Anorexia nervosa | ICD-10: F50.0, F50.1; ICD-9: 307B/A; ICD-8: 306.5 | National Hospital Registries (inpatient & outpatient) |
| Michels et al., 2004 | Anorexia nervosa | ICD-9: 307B; ICD-8: 306.50; ICD-7: 316.99, 784.09 | Swedish Inpatient Registry |
| Momen et al., 2020 | Schizophrenia, mood disorders, anxiety and stress-related disorders, eating disorders, autism, intellectual disability | Schizophrenia: ICD-10: F20-29; ICD-8: 295.x9, 296.89, 297.x9, 298.29-298.99, 299.04, 299.05, 299.09, 301.83  Mood disorders: ICD-10: F30-39; ICD-8: 296.x9 (excluding 296.89), 298.09, 298.19, 300.49, 301.19  Anxiety and stress-related disorders: ICD-10: F40-48; ICD-8: 300.x9 (excluding 300.49), 305.x9, 305.68, 307.99  Eating disorders: ICD-10: F50; ICD-8: 305.60, 306.50, 306.58, 306.59  Autism: ICD-10: F84.0, F84.5, F84.8, F84.9; ICD-9: 299A, 299B, 299X  Intellectual disability: ICD-10: F70-79; ICD-8: 311.xx, 312.xx, 313.xx, 314.xx, 315.xx | Danish Psychiatric Central Research Register (inpatient & outpatient) |
| Mortensen, 1989 | Schizophrenia | Not reported | Psychiatric inpatients in Denmark  on a census day in 1957 |
| Pettersson et al., 2020 | Schizophrenia | ICD-10: F20-29, ICD-9 and ICD-8: 295, 297, 298 | National Patient Register (inpatient) |
| Tian et al., 2022 | Stress-related disorders | ICD-10: F43; ICD-9: 308, 309, ICD-8: 307 | Swedish National Patient Register (inpatient & outpatient) |
| Wium-Andersen et al., 2021 | Mood disorders | Bipolar disorder: ICD-10: F30-31  Major depressive disorder: F32-33  Other affective disorders: F34, F38, F39 | Danish Psychiatric Central Register and Danish National Patient Registry (inpatient & outpatient) |

ICD=International Classification of Diseases

# **Supplementary Table 4:** Results from models including total study population, by type of mental illness and cancer

| **Author**  **(Year)** | **Measure of association** | **Model adjustments** | **All cancer** | **Breast** | **Colon** | **Rectum** | **Kidney** | **Lung** | **Pancreas** | **Prostate** | **Urinary bladder** | **Uterine** |
| --- | --- | --- | --- | --- | --- | --- | --- | --- | --- | --- | --- | --- |
| **Schizophrenia** | | | | | | | | | | | | |
| Dalton  (2003) | RR  (95% CI) | Age, calendar period, age at birth of first child, number of live births |  | 0.90  (0.71-1.12) |  |  |  |  |  |  |  |  |
| Dalton  (2005) | SIR  (95% CI) | Standardized by age, sex, calendar period |  | 1.20  (1.05-1.38) |  |  |  |  |  | 0.56  (0.37-0.81) |  | 0.86  (0.59-1.20) |
| Ji  (2013) | SIR  (95% CI) | Standardized by 5-year age group, gender, 5-year time period, socioeconomic status, residential area, comorbidities; further by age at first birth and parity for female reproductive cancers | 1.00  (0.97-1.03) | 1.47  (1.38-1.56) | 1.00  (0.90-1.11) | 0.82  (0.70-0.95) | 1.09  (0.93-1.27) | 1.01  (0.92-1.11) | 1.00  (0.84-1.18) | 0.53  (0.48-0.59) | 0.73  (0.63-0.85) | 1.34  (1.17-1.53) |
| Lichtermann (2001) | SIR  (95% CI) | Standardized by duration of follow-up, 5-year age group | 1.17  (1.09-1.25) | 1.15  (0.98-1.34) | 0.86  (0.55-1.27) | 0.35  (0.13-0.75) | 1.30  (0.85-1.91) | 2.17  (1.78-2.60) | 1.16  (0.67-1.84) | 0.49  (0.20-1.00) |  | 1.75  (1.19-2.48) |
| Lu  (2020) | OR  (95% CI) | Birth year, age, education, region, parity, pre-existing psychiatric disorder, obesity |  | 1.39  (1.28–1.52) |  |  |  |  |  |  |  |  |
| Momen (2020) | HR  (95% CI) | Age at follow-up, sex, birth date, other mental disorders diagnosed before mental disorder of interest | 1.05  (1.03-1.08) |  |  |  |  |  |  |  |  |  |
| **Author**  **(Year)** | **Measure of association** | **Model adjustments** | **All cancer** | **Breast** | **Colon** | **Rectum** | **Kidney** | **Lung** | **Pancreas** | **Prostate** | **Urinary bladder** | **Uterine** |
| Mortensen (1989) | SIR  (95% CI) | Standardized by age | 0.84  (0.79-0.89) | 1.16  (0.96-1.38) | 0.95  (0.77-1.16) | 0.75  (0.57-0.98) | 1.09  (0.76-1.51) | 0.32  (0.23-0.43) | 0.95  (0.68-1.30) | 0.54  (0.38-0.73) | 0.76  (0.55-1.01) | 0.78  (0.49-1.18) |
| Pettersson (2020) | IRR  (95% CI) | Sex, calendar year at follow-up, age at follow-up | 1.02  (0.91-1.13) | 1.19  (1.12-1.26) | 0.94  (0.87-1.01) |  |  | 1.42  (1.28-1.58) | 1.10  (1.01-1.21) | 0.66  (0.55-0.79) |  |  |
| **Mood disorders** | | | | | | | | | | | | |
| Dalton (2002) | SIR  (95% CI) | Standardized by age, sex, calendar year | BP: 0.98  (0.95-1.01)  Other: 1.14  (1.10-1.17) | BP: 1.04  (0.97-1.11)  Other: 1.06  (0.98-1.15) | BP: 0.93  (0.85-1.03)  Other: 1.16  (1.03-1.30) | BP: 0.79  (0.68-0.92)  Other: 0.83  (0.69-1.00) | BP: 1.04  (0.87-1.23)  Other: 1.19  (0.97-1.45) | BP: 1.00  (0.93-1.09)  Other: 1.59  (1.47-1.72) | BP: 1.05  (0.89-1.22)  Other: 1.52  (1.27-1.79) | BP: 0.96  (0.83-1.11)  Other: 1.02  (0.84-1.23) | BP: 1.04  (0.92-1.17)  Other: 1.23  (1.07-1.41) | BP: 0.92  (0.79-1.07)  Other: 0.87  (0.72-1.05) |
| Momen (2020) | HR  (95% CI) | Age at follow-up, sex, birth date, other mental disorders diagnosed before mental disorder of interest | 1.03 (1.02-1.05) |  |  |  |  |  |  |  |  |  |
| Wium-Andersen (2021) | HR  (95% CI) | Age, gender, calendar time, marital status, education, somatic and psychotropic medication use during the 2 years before start of follow-up |  | BD: 1.07  (0.91-1.27) MDD: 0.93  (0.86-1.00) Other: 1.01  (0.85-1.20) | ^+^BD: 0.87  (0.73-1.05) ^+^MDD: 0.99  (0.92-1.07) ^+^Other: 1.12  (0.94-1.34) | |  | BD: 1.07  (0.92-1.26) MDD: 1.12  (1.04-1.20) Other: 1.07  (0.90-1.27) |  | BD: 0.87  (0.71-1.08) MDD: 0.91  (0.83-0.99) Other: 0.87  (0.66-1.14) |  |  |
| **Author**  **(Year)** | **Measure of association** | **Model adjustments** | **All cancer** | **Breast** | **Colon** | **Rectum** | **Kidney** | **Lung** | **Pancreas** | **Prostate** | **Urinary bladder** | **Uterine** |
| **Anxiety and stress-related disorders** | | | | | | | | | | | | |
| Gradus (2015) | SIR  (95% CI) | Standardized by sex, 5-year age group, 5-year calendar period | 1.00  (0.88-1.20) | 1.20  (0.82-1.70) | 0.59  (0.19-1.40) |  |  |  |  |  |  | 1.40 (0.46-3.30) |
| Momen (2020) | HR  (95% CI) | Age at follow-up, sex, birth date, other mental disorders diagnosed before mental disorder of interest | 1.13  (1.11-1.15) |  |  |  |  |  |  |  |  |  |
| Tian  (2022) | HR  (95% CI) | Birth year, sex, education, family income, marital status, family history of cancer, Charlson's comorbidity index, history of substance use disorders | 1.03  (1.01-1.06) | 1.05  (0.99-1.12) | ^+^0.96  (0.87-1.05) | |  | 1.25  (1.15-1.37) | 1.50  (1.28-1.75) | 0.89  (0.82-0.96) |  |  |
| **Eating disorders** | | | | | | | | | | | | |
| Karamanis (2014) | SIR  (95% CI) | Standardized by 5-year age group, calendar year |  | 0.70  (0.40-1.10) |  |  |  |  |  |  |  |  |
| Mellemkjaer (2001) | SIR  (95% CI) | Standardized by age, sex, calendar year |  | 0.80  (0.30-1.70) |  |  |  |  |  |  |  |  |
| Mellemkjaer  (2015) | IRR  (95% CI) | Sex, birth year, place of residence, current age. Also, age at first childbirth and parity for females |  | 0.60  (0.50-0.80) |  |  |  |  |  | 0.50  (0.10-3.40) |  |  |
| Michels (2004) | SIR  (95% CI) | Standardized by age group (5-year), calendar year |  | 0.47  (0.19-0.97) |  |  |  |  |  |  |  |  |
| **Author**  **(Year)** | **Measure of association** | **Model adjustments** | **All cancer** | **Breast** | **Colon** | **Rectum** | **Kidney** | **Lung** | **Pancreas** | **Prostate** | **Urinary bladder** | **Uterine** |
| Momen (2020) | HR  (95% CI) | Age at follow-up, sex, birth date, other mental disorders diagnosed before mental disorder of interest | 1.26  (1.14-1.39) |  |  |  |  |  |  |  |  |  |
| **Neurodevelopmental disorders** | | | | | | | | | | | | |
| Liu  (2022)* | OR  (95% CI) | Birth year, sex, parental age at delivery, and parental history of psychiatric disorders or cancer at delivery | 1.30  (1.20-1.50) |  | 1.80  (0.70-3.60) |  | 1.90  (1.00-3.30) |  | 6.20  (0.60-27.50) |  | 2.40  (0.30-8.90) |  |
| Liu  (2021)** | HR  (95% CI) | Age at follow-up, birth year, sex, maternal and paternal age at delivery, maternal/paternal history of psychiatric disorders or cancer at delivery | 1.57  (1.35-1.82) | 0.70  (0.30-1.60) | 2.00  (1.00-4.10) | 2.00  (0.50-8.00) | 4.40  (2.00-9.80) | 1.10  (0.10-7.50) | 6.00  (1.50-24.80) |  |  | 11.70  (1.50-90.70) |
| Momen (2020)* | HR  (95% CI) | Age at follow-up, sex, birth date, and other mental disorders diagnosed before mental disorder of interest | 0.97  (0.90-1.04) |  |  |  |  |  |  |  |  |  |

BP=bipolar disorder; MDD=major depressive disorder; Other=other affective disorders.

^+^Model result for colorectal cancer

*Exposure=autism

**Exposure=intellectual disability

# **Supplementary Table 5:** Sex-stratified model results by type of mental illness and cancer

| **Author**  **(Year)** | **Measure of association** | **Model adjustments** | **All cancer** | | **Colon** | | **Kidney** | | | **Lung** | | | | | **Pancreas** | | | | | | **Rectum** | | | | | | **Urinary bladder** | | | |
| --- | --- | --- | --- | --- | --- | --- | --- | --- | --- | --- | --- | --- | --- | --- | --- | --- | --- | --- | --- | --- | --- | --- | --- | --- | --- | --- | --- | --- | --- | --- |
|  |  |  | **Male** | **Female** | **Male** | **Female** | **Male** | **Female** | | **Male** | | **Female** | | | **Male** | | | **Female** | | | **Male** | | | **Female** | | | **Male** | | **Female** | |
| **Schizophrenia** | | | | | | | | | | | | | | | | | | | | | | | | | | | | | | |
| Crump  (2013) | HR  (95% CI) | Age, marital status, education, employment status, income | 0.84 (0.73-0.96) | 1.09 (0.97-1.23) |  |  |  |  | |  | |  | | |  | | |  | | |  | | |  | | |  | |  | |
| Dalton  (2005) | SIR  (95% CI) | Standardized by age, sex, calendar period | 0.85 (0.78-0.93) | 1.03 (0.96-1.11) | 0.93 (0.66-1.28) | 0.96 (0.72-1.25) | 1.00 (0.59-1.59) | 1.23 (0.74-1.92) | | 0.82 (0.65-1.02) | | 1.17 (0.90-1.49) | | | 0.77 (0.40-1.35) | | | 0.64 (0.33-1.12) | | | 0.62 (0.37-0.98) | | | 1.22 (0.83-1.72) | | | 0.78 (0.56-1.07) | | 0.85 (0.48-1.37) | |
| Ji  (2013) | SIR  (95% CI) | Standardized by 5-year age group, gender, 5-year time period, socioeconomic status, residential area, comorbidities; further by age at first birth and parity for female reproductive cancers | 0.79 (0.75-0.82) | 1.20 (1.15-1.24) | 0.89 (0.76-1.04) | 1.04 (0.90-1.19) | 1.18 (0.96-1.43) | 0.97 (0.73-1.26) | | 0.93 (0.82-1.05) | | 1.22 (1.05-1.41) | | | 1.00 (0.78-1.26) | | | 1.00 (0.77-1.27) | | | 0.70 (0.56-0.87) | | | 0.93 (0.74-1.15) | | | 0.68 (0.57-0.82) | | 0.81 (0.60-1.07) | |
| Lichtermann  (2001) | SIR  (95% CI) | Standardized by duration of follow-up, 5-year age group | 1.25 (1.11-1.38) | 1.12 (1.01-1.22) |  |  |  |  | |  | |  | | |  | | |  | | |  | | |  | | |  | |  | |
| Momen  (2020) | HR  (95% CI) | Age at follow-up, sex, birth date, and other mental disorders diagnosed before mental disorder of interest | 0.96 (0.92-0.99) | 1.12 (1.09-1.16) |  |  |  |  | |  | |  | | |  | | |  | | |  | | |  | | |  | |  | |
| Mortensen  (1989) | SIR  (95% CI) | Standardized by age | 0.69 (0.62-0.76) | 0.99 (0.91-1.08) | 0.77 (0.53-1.07) | 1.09 (0.84-1.39) | 0.86 (0.49-1.40) | 1.38 (0.84-2.13) | | 0.34 (0.23-0.47) | | 0.25 (0.09-0.54) | | | 0.50 (0.25-0.89) | | | 1.44 (0.96-2.07) | | | 0.71 (0.48-1.01) | | | 0.81 (0.53-1.19) | | | 0.65 (0.44-0.92) | | 1.08 (0.63-1.72) | |
| Pettersson  (2020) | IRR  (95% CI) | Sex, calendar year at follow-up, age at follow-up | 0.93 (0.89-0.98) | 1.11 (1.08-1.15) | 0.91 (0.81-1.01) | 0.97 (0.89-1.05) |  |  | | 1.49 (1.35-1.63) | | 1.40 (1.27-1.55) | | | 1.22 (1.07-1.39) | | | 1.03 (0.92-1.16) | | |  | | |  | | |  | |  | |
| **Author**  **(Year)** | **Measure of association** | **Model adjustments** | **All cancer** | | **Colon** | | **Kidney** | | | | **Lung** | | | | | | **Pancreas** | | | | | | **Rectum** | | | | | **Urinary bladder** | | |
|  |  |  | **Male** | **Female** | **Male** | **Female** | **Male** | | **Female** | | **Male** | | | **Female** | | | **Male** | | | **Female** | | | **Male** | | | **Female** | | **Male** | | **Female** |
| **Mood disorders** | | | | | | | | | | | | | | | | | | | | | | | | | | | | | | |
| Crump  (2013) (BP) | HR  (95% CI) | Age, marital status, education, employment status, income | 0.99 (0.87-1.12) | 1.08 (0.98-1.20) |  |  |  | |  | |  | | |  | | |  | | |  | | |  | | |  | |  | |  |
| Momen  (2020) | HR  (95% CI) | Age at follow-up, sex, birth date, other mental disorders diagnosed before mental disorder of interest | 1.05 (1.03-1.08) | 1.02 (1.00-1.04) |  |  |  | |  | |  | | |  | | |  | | |  | | |  | | |  | |  | |  |
| **Anxiety and stress-related disorders** | | | | | | | | | | | | | | | | | | | | | | | | | | | | | | |
| Gradus  (2015) | SIR  (95% CI) | Standardized by sex, 5-year age group, 5-year calendar period | 1.00 (0.79-1.30) | 1.00 (0.84-1.30) |  |  |  | |  | |  | | |  | | |  | | |  | | |  | | |  | |  | |  |
| Momen  (2020) | HR  (95% CI) | Age at follow-up, sex, birth date, other mental disorders diagnosed before mental disorder of interest | 1.06 (1.03-1.08) | 1.18 (1.15-1.20) |  |  |  | |  | |  | | |  | | |  | | |  | | |  | | |  | |  | |  |
| Tian  (2022) | HR  (95% CI) | Birth year, sex, education, family income, marital status, family history of cancer, Charlson's comorbidity index, history of substance use disorders | 1.02 (0.98-1.06) | 1.04 (1.01-1.07) |  |  |  | |  | |  | | |  | | |  | | |  | | |  | | |  | |  | |  |
| **Eating disorders** | | | | | | | | | | | | | | | | | | | | | | | | | | | | | | |
| Karamanis  (2014) | SIR  (95% CI) | Standardized by 5-year age group, calendar year |  | 1.10 (0.80-1.30) |  |  |  | |  | |  | |  | | |  | | |  | | |  | | |  | | |  | |  |
| **Author**  **(Year)** | **Measure of association** | **Model adjustments** | **All cancer** | | **Colon** | | **Kidney** | | | | **Lung** | | | | | **Pancreas** | | | | | | **Rectum** | | | | | | **Urinary bladder** | | |
|  |  |  | **Male** | **Female** | **Male** | **Female** | **Male** | | **Female** | | **Male** | | **Female** | | | **Male** | | | **Female** | | | **Male** | | | **Female** | | | **Male** | | **Female** |
| Mellemkjaer  (2001) | SIR  (95% CI) | Standardized by age, sex, calendar year | 1.30 (0.20-4.70) | 0.80 (0.52-1.18) |  |  |  | |  | |  | | 2.20 (0.50-6.40) | | |  | | | 4.40 (0.10-24.20) | | |  | | |  | | |  | |  |
| Mellemkjaer  (2015) | IRR  (95% CI) | Sex, birth year, place of residence, current age. Also age at first childbirth and parity for females | Overall: 1.08 (0.71-1.66)  Sw: 1.09 (0.60-1.97)  De: 1.18 (0.57-2.43)  Fi:  0.91 (0.28-2.94) | Overall: 0.97 (0.88-1.08)  Sw: 0.94 (0.80-1.10)  De: 0.99 (0.84-1.16)  Fi:  1.08 (0.78-1.50) | 0.50 (0.10-3.70)^+^ | 1.20 (0.80-1.80)^+^ | 4.20 (0.40-40.90) | | 1.00 (0.30-3.30) | | 0.90 (0.10-6.80) | | 1.60 (1.10-2.40) | | | 3.80 (0.40-34.30) | | | 1.80 (0.80-4.30) | | |  | | |  | | |  | |  |
| Michels  (2004) | SIR  (95% CI) | Standardized by age group (5-year), calendar year |  | 0.92 (0.69-1.21) |  |  |  | |  | |  | |  | | |  | | |  | | |  | | |  | | |  | |  |
| Momen  (2020) | HR  (95% CI) | Age at follow-up, sex, birth date, other mental disorders diagnosed before mental disorder of interest | 0.75 (0.45-1.27) | 1.29 (1.17-1.43) |  |  |  | |  | |  | |  | | |  | | |  | | |  | | |  | | |  | |  |
| **Neurodevelopmental disorders** | | | | | | | | | | | | | | | | | | | | | | | | | | | | | | |
| Momen (2020)** | HR  (95% CI) | Age at follow-up, sex, birth date, and other mental disorders diagnosed before mental disorder of interest | 0.81 (0.73-0.90) | 1.15 (1.04-1.27) |  |  |  | |  | |  | | |  | |  | | |  | | |  | | |  | | |  | |  |

Sw=Sweden; De=Denmark; Fi=Finland

**Exposure=intellectual disability

^+^Model result for colorectal cancer

# **Supplementary Table 6:** Results from Egger’s test for publication bias in the meta-analyses

| **Meta analysis** | **p-value from Egger’s test** |
| --- | --- |
| Meta-analysis 1 | 0.848 |
| Meta-analysis 2 | 0.932 |

Meta-analysis 1: meta-analysis of the most recent studies from each country using standardized incidence rates (SIR) to measure the association between schizophrenia diagnoses and the risk of each cancer type.

Meta-analysis 2: Meta-analysis of most recent studies from each country investigating the association between schizophrenia diagnoses and the risk of each cancer type, regardless of measure of association used.

# **Supplementary Figure 1:** Meta-analyses of the most recent studies from each country investigating the association between schizophrenia diagnoses and the risk of each cancer type, regardless of measure of association used

Pooled estimates combine standardized incidence ratios (SIR), odds ratios (OR), hazard ratios (HR), and incidence rate ratios (IRR).

# **PRISMA Checklist**

| **Section and Topic** | **Item #** | **Checklist item** | **Location where item is reported** |
| --- | --- | --- | --- |
| **TITLE** | | |  |
| Title | 1 | Identify the report as a systematic review. | Page 1 |
| **ABSTRACT** | | |  |
| Abstract | 2 | See the PRISMA 2020 for Abstracts checklist. | Page 3 |
| **INTRODUCTION** | | |  |
| Rationale | 3 | Describe the rationale for the review in the context of existing knowledge. | Pages 4-5 |
| Objectives | 4 | Provide an explicit statement of the objective(s) or question(s) the review addresses. | Page 5 |
| **METHODS** | | |  |
| Eligibility criteria | 5 | Specify the inclusion and exclusion criteria for the review and how studies were grouped for the syntheses. | Pages 5-6 |
| Information sources | 6 | Specify all databases, registers, websites, organisations, reference lists and other sources searched or consulted to identify studies. Specify the date when each source was last searched or consulted. | Page 6 |
| Search strategy | 7 | Present the full search strategies for all databases, registers and websites, including any filters and limits used. | Supplementary Table 1 |
| Selection process | 8 | Specify the methods used to decide whether a study met the inclusion criteria of the review, including how many reviewers screened each record and each report retrieved, whether they worked independently, and if applicable, details of automation tools used in the process. | Page 6 |
| Data collection process | 9 | Specify the methods used to collect data from reports, including how many reviewers collected data from each report, whether they worked independently, any processes for obtaining or confirming data from study investigators, and if applicable, details of automation tools used in the process. | Page 7 |
| Data items | 10a | List and define all outcomes for which data were sought. Specify whether all results that were compatible with each outcome domain in each study were sought (e.g. for all measures, time points, analyses), and if not, the methods used to decide which results to collect. | Pages 6-7 |
|  | 10b | List and define all other variables for which data were sought (e.g. participant and intervention characteristics, funding sources). Describe any assumptions made about any missing or unclear information. | Pages 6-7 |
| Study risk of bias assessment | 11 | Specify the methods used to assess risk of bias in the included studies, including details of the tool(s) used, how many reviewers assessed each study and whether they worked independently, and if applicable, details of automation tools used in the process. | Page 7 |
| Effect measures | 12 | Specify for each outcome the effect measure(s) (e.g. risk ratio, mean difference) used in the synthesis or presentation of results. | Page 7 |
| Synthesis methods | 13a | Describe the processes used to decide which studies were eligible for each synthesis (e.g. tabulating the study intervention characteristics and comparing against the planned groups for each synthesis (item #5)). | Page 7 |
|  | 13b | Describe any methods required to prepare the data for presentation or synthesis, such as handling of missing summary statistics, or data conversions. | Pages 7-8 |
|  | 13c | Describe any methods used to tabulate or visually display results of individual studies and syntheses. | Page 7 |
|  | 13d | Describe any methods used to synthesize results and provide a rationale for the choice(s). If meta-analysis was performed, describe the model(s), method(s) to identify the presence and extent of statistical heterogeneity, and software package(s) used. | Pages 7-8 |
|  | 13e | Describe any methods used to explore possible causes of heterogeneity among study results (e.g. subgroup analysis, meta-regression). | Page 7 |
|  | 13f | Describe any sensitivity analyses conducted to assess robustness of the synthesized results. | Page 7 |
| Reporting bias assessment | 14 | Describe any methods used to assess risk of bias due to missing results in a synthesis (arising from reporting biases). | Page 8 |
| Certainty assessment | 15 | Describe any methods used to assess certainty (or confidence) in the body of evidence for an outcome. | NA |
| **RESULTS** | | |  |
| Study selection | 16a | Describe the results of the search and selection process, from the number of records identified in the search to the number of studies included in the review, ideally using a flow diagram. | Page 8, Figure 1 |
|  | 16b | Cite studies that might appear to meet the inclusion criteria, but which were excluded, and explain why they were excluded. | Page 6 |
| Study characteristics | 17 | Cite each included study and present its characteristics. | Page 8, Table 1 |
| Risk of bias in studies | 18 | Present assessments of risk of bias for each included study. | Page 9, Supplementary Table 2 |
| Results of individual studies | 19 | For all outcomes, present, for each study: (a) summary statistics for each group (where appropriate) and (b) an effect estimate and its precision (e.g. confidence/credible interval), ideally using structured tables or plots. | Figures 2 and 3, Supplementary Table 4, Supplementary Figure 1 |
| Results of syntheses | 20a | For each synthesis, briefly summarise the characteristics and risk of bias among contributing studies. | Pages 9-12 |
|  | 20b | Present results of all statistical syntheses conducted. If meta-analysis was done, present for each the summary estimate and its precision (e.g. confidence/credible interval) and measures of statistical heterogeneity. If comparing groups, describe the direction of the effect. | Figures 2 and 3, Supplementary Figure 1 |
|  | 20c | Present results of all investigations of possible causes of heterogeneity among study results. | Figure 3, Supplementary Figure 1 |
|  | 20d | Present results of all sensitivity analyses conducted to assess the robustness of the synthesized results. | Figure 3, Supplementary Figure 1 |
| Reporting biases | 21 | Present assessments of risk of bias due to missing results (arising from reporting biases) for each synthesis assessed. | Page 10, Supplementary Table 6 |
| Certainty of evidence | 22 | Present assessments of certainty (or confidence) in the body of evidence for each outcome assessed. | NA |
| **DISCUSSION** | | |  |
| Discussion | 23a | Provide a general interpretation of the results in the context of other evidence. | Pages 12-14 |
|  | 23b | Discuss any limitations of the evidence included in the review. | Pages 14-15 |
|  | 23c | Discuss any limitations of the review processes used. | Page 14 |
|  | 23d | Discuss implications of the results for practice, policy, and future research. | Pages 12-15 |
| **OTHER INFORMATION** | | |  |
| Registration and protocol | 24a | Provide registration information for the review, including register name and registration number, or state that the review was not registered. | Page 5 |
|  | 24b | Indicate where the review protocol can be accessed, or state that a protocol was not prepared. | Page 5 |
|  | 24c | Describe and explain any amendments to information provided at registration or in the protocol. | Page 5 |
| Support | 25 | Describe sources of financial or non-financial support for the review, and the role of the funders or sponsors in the review. | Pages 8 & 16 |
| Competing interests | 26 | Declare any competing interests of review authors. | Page 16 |
| Availability of data, code and other materials | 27 | Report which of the following are publicly available and where they can be found: template data collection forms; data extracted from included studies; data used for all analyses; analytic code; any other materials used in the review. | Page 8 |

*From:* Page MJ, McKenzie JE, Bossuyt PM, Boutron I, Hoffmann TC, Mulrow CD, et al. The PRISMA 2020 statement: an updated guideline for reporting systematic reviews. BMJ 2021;372:n71. doi: 10.1136/bmj.n71. This work is licensed under CC BY 4.0. To view a copy of this license, visit <https://creativecommons.org/licenses/by/4.0/>
